# Short-term visual acuity outcomes and prognostic factors following 25-gauge pars plana vitrectomy for intraocular foreign body extraction

**DOI:** 10.3389/fmed.2026.1746762

**Published:** 2026-04-01

**Authors:** Yanyan Liang, Xi Lv, Jialin Niu, Shifeng Zhang, Pei Feng, Zhaohui Xiong, Ye Yang

**Affiliations:** 1 Department of Ophthalmology, The First Hospital of Hebei Medical University, Shijiazhuang, Hebei, China; 2 Department of Ophthalmology, Hebei General Hospital, Shijiazhuang, Hebei, China; 3 Department of Ophthalmology, The Forth Hospital of Hebei Medical University, Shijiazhuang, Hebei, China

**Keywords:** 25G minimally invasive vitrectomy, endophthalmitis, intraocular foreign bodies, macula, postoperative visual acuity

## Abstract

**Objective:**

To characterize the clinical presentation of intraocular foreign body (IOFB) injury and evaluate the short-term visual acuity outcomes following 25-gauge pars plana vitrectomy (PPV), with identification of independent predictors of postoperative visual recovery.

**Methods:**

A retrospective study was conducted on 198 patients (198 eyes) diagnosed with IOFB who underwent 25G minimally invasive PPV at the Department of Ophthalmology, First Hospital of Hebei Medical University, between January 1, 2020, and December 31, 2024. Clinical characteristics of the IOFB, therapeutic efficacy of PPV, and best corrected visual acuity (BCVA) were analyzed to identify factors influencing postoperative visual acuity. Furthermore, receiver operating characteristic (ROC) curve analysis was performed to validate the predictive accuracy of the model for postoperative visual outcomes.

**Results:**

Among the 198 patients, most were young and middle-aged males residing in rural areas. Metal IOFB were the predominant type, accounting for 141 eyes (71.2%). The majority of these foreign bodies entered through Zone I (157 eyes, 79.3%), with a complete intraoperative removal rate (100%). Preoperatively, BCVA was ≥0.1 in 81 eyes and <0.1 in 117 eyes. Postoperatively, BCVA improved to ≥0.1 in 126 eyes, while it remained below 0.1 in 72 eyes. This improvement in BCVA after surgery was statistically significant (*p* < 0.001). Multivariate logistic regression analysis revealed that poor preoperative BCVA, macular involvement by the foreign body and coexisting endophthalmitis were independent risk factors for suboptimal postoperative visual outcomes. A composite predictive model integrating these three variables exhibited strong discriminatory performance, with an area under the AUC of 0.797 (95% confidence interval: 0.735–0.859), with a sensitivity of 94.4% and specificity of 55.6%, indicating high predictive accuracy.

**Conclusion:**

25G minimally invasive PPV effectively improves visual acuity in the majority of patients with IOFB. Preoperative poor BCVA, macular impaction of foreign bodies and coexisting endophthalmitis are significant independent risk factors for short-term suboptimal postoperative visual outcomes.

## 1 Introduction

Intraocular foreign body (IOFB) constitute a common and clinically significant ophthalmic emergency. Epidemiological studies indicate that the incidence of IOFB in China ranges from 18% to 41%, with approximately 72.28% involving the posterior segment of the eye (1). These injuries predominantly occur in middle-aged males and are typically associated with severe ocular trauma and an unfavorable visual prognosis, often resulting in considerable economic and psychological burdens for both patients and their families. 25G minimally invasive vitrectomy has been widely adopted in the management of fundus disorders due to its advantages, including minimal surgical trauma, reduced postoperative inflammation, and accelerated recovery. As microsurgical techniques continue to advance, this procedure has become the preferred intervention for IOFB removal (2). However, the factors influencing postoperative visual acuity remain a subject of ongoing debate. To address this issue, we conducted a retrospective analysis of clinical data from 198 patients with intraocular foreign bodies who underwent 25G minimally invasive vitrectomy, aiming to identify key determinants of visual prognosis and characterize the clinical features of such injuries.

## 2 Patients and methods

### 2.1 Research subjects

A retrospective observational study was carried out on 198 patients (198 eyes) who received treatment at the Department of Ophthalmology, the First Hospital of Hebei Medical University, between January 1, 2020, and December 31, 2024. All participants sustained penetrating ocular injuries accompanied by intraocular foreign bodies and underwent 25G minimally invasive vitrectomy. Inclusion criteria: A documented history of ocular trauma; confirmed presence of distinct intraocular foreign bodies located within the vitreous cavity, on the retinal surface, or subretinally, as verified by orbital CT, B-mode ultrasonography, or fundus photography; and completion of 25-gauge minimally invasive vitrectomy during hospitalization. Exclusion criteria: Patients who are unable to comply with surgical procedures due to medical conditions or behavioral factors that may compromise procedural safety or outcomes. This study was approved by the Institutional Review Board (IRB) of the First Hospital of Hebei Medical University (approval number: 20221107), and written informed consent was obtained from all participants prior to diagnostic and surgical procedures.

### 2.2 Methods

#### 2.2.1 Surgical techniques

Upon admission, all patients underwent comprehensive preoperative evaluations and received standardized medical management. Routine diagnostic assessments included orbital CT, BCVA testing, slit-lamp biomicroscopy, and fundus examination. Surgical procedures were performed by an experienced vitreoretinal surgical team comprising consultants or associate senior-level physicians. The intervention consisted of a 25-gauge minimally invasive vitrectomy combined with IOFB removal. For patients presenting with inadequate wound closure, primary debridement and suturing were initially performed, followed by secondary foreign body extraction via a three-port pars plana incision between 3 and 13 days postoperatively. Based on the precise anatomical location of the foreign body, the surgical approach was selected from the original injury tract, corneal incision, or pars plana incision. Under direct visualization, the foreign body was meticulously dissected in layers and safely extracted using either magnetic retrieval or microsurgical instruments. In cases of severe lens opacification, concurrent lens extraction was carried out, with intraocular lens implantation scheduled at a later stage according to individual clinical conditions. When endophthalmitis was suspected, prophylactic intravitreal antibiotics, including vancomycin (1 mg/mL) and ceftazidime (2 mg/mL), were administered through the intraoperative irrigation solution. During surgery, retinal laser photocoagulation was conducted following retinal flattening with sterile air or perfluorocarbon liquid, depending on the presence of retinal injury or detachment. At the conclusion of the procedure, the vitreous cavity was filled with sterile air, balanced salt solution, or silicone oil to ensure stable retinal reattachment.

#### 2.2.2 Postoperative management

Postoperatively, patients received localized therapeutic interventions, including anti-inflammatory, anti-infective, and mydriatic treatments. Empirical or culture-guided antibiotic therapy, in combination with glucocorticoid administration, was implemented based on clinical assessment and microbiological evidence.

#### 2.2.3 Outcome measures

Patient demographic and clinical parameters, including age, gender, time interval between injury occurrence and medical presentation, BCVA at admission, site of foreign body entry into the globe, concomitant ocular pathologies, as well as the characteristics (type, size, and location of impaction) of the foreign body, and postoperative BCVA were prospectively collected and systematically analyzed.

The wound locations in cases of open globe injuries were categorized into three anatomically defined zones based on their spatial distribution: Zone I encompassed the corneal region, including the corneal limbus; Zone II extended from the corneal limbus to a maximum of 5 mm posterior along the sclera; and Zone III referred to the scleral area located beyond 5 mm posterior to the corneal limbus.

Patients were stratified by BCVA measured at 1 week postoperatively, using a decimal BCVA of 0.1 as the prespecified dichotomous outcome threshold: those achieving BCVA ≥ 0.1 were categorized as having a favorable visual outcome, and those with BCVA < 0.1 as having an unfavorable visual outcome. This classification framework was employed to identify potential prognostic factors associated with visual recovery after surgical management.

### 2.3 Statistical analysis

All statistical analyses were carried out using SPSS 24.0 software (IBM Corporation, Armonk, NY, United States). The following analytical strategies were employed to identify clinical determinants of suboptimal short-term visual recovery after 25-gauge pars plana vitrectomy for intraocular foreign body extraction:

(1) To assess categorical transitions in best-corrected visual acuity (BCVA) from preoperative baseline to 1 week postoperatively, McNemar’s test was performed—specifically designed for hypothesis testing of discordant paired nominal outcomes within the same patient cohort.
(2) Univariate associations between categorical covariates and the primary outcome (suboptimal visual recovery, defined as BCVA < 0.1 at 1 week postoperatively) were evaluated using Pearson’s chi-square test when all expected cell counts were ≥ 5; Fisher’s exact test was applied otherwise. A multivariable logistic regression model was then fitted to identify independent predictors of the outcome. Age, sex, and intraocular foreign body (IOFB) location were prespecified as mandatory adjustment variables grounded in pathophysiological plausibility and empirical support from prior literature, and retained in the final model regardless of statistical significance to ensure robust control for potential confounding. Remaining candidate variables underwent backward stepwise elimination based on the Wald statistic (*p*-to-remove > 0.10).
(3) A clinical prediction model was developed incorporating only the independent predictors retained in the final multivariable model. Model-predicted probabilities of suboptimal visual recovery served as the test variable, and the binary outcome—failure to achieve BCVA ≥ 0.1 at 1 week postoperatively—was used as the state variable for receiver operating characteristic (ROC) curve analysis. Discriminatory accuracy was quantified by the area under the ROC curve (AUC), with the optimal diagnostic threshold determined by maximizing the Youden index. Sensitivity and specificity were reported at this threshold.

## 3 Results

### 3.1 Baseline characteristics

A total of 198 patients (198 eyes) were enrolled in this study, consisting of 188 male and 10 female individuals. During surgical procedures, foreign bodies were extracted from the pars plana of the ciliary body in 86 eyes (43.4%), from the original corneal wound in 30 eyes (15.2%), and from the corneal limbus in 82 eyes (41.4%). The baseline demographic and clinical characteristics of the patients, including age, etiology of injury, and intraoperative ocular findings, are summarized in Table 1.

**Table 1:** Baseline characteristics of patients.

Characteristics	Number of cases	Proportion (%)
Gender		
Male	188	94.9
Female	10	5.1
Eye laterality		
Left	101	51
Right	97	49
Age		
<18	10	5.1
18–60	174	87.9
>60	14	7.1
Regional sources		
Rural	148	74.7
Urban	50	25.3
Types of foreign bodies		
Metal	141	71.2
Stone	16	8.1
China	12	6.1
Plants	14	7.1
Explosion	6	3.0
Other*	9	4.5
Postoperative filling materials		
Silicone oil	146	73.7
Gas	28	14.1
Perfusate	24	12.1

*Preoperative orbital CT demonstrated a high-density opacity within the vitreous cavity. Given the patient’s history of ocular trauma and the minute dimensions of the foreign body, extraction via the vitrectomy probe was undertaken. However, definitive characterization of the foreign body’s composition remained indeterminate.

### 3.2 Complications

Among the eyes that underwent surgical intervention, traumatic cataract was diagnosed in 160 eyes (80.8%), vitreous hemorrhage was observed in 70 eyes (35.4%), retinal detachment was identified in 46 eyes (23.2%), and endophthalmitis complicated the condition in 23 eyes (11.6%).

### 3.3 Short-term surgical efficacy

In all 198 cases of IOFBs, complete removal was successfully achieved through vitrectomy surgery, resulting in a 100% extraction rate. Preoperatively, BCVA was less than 0.1 in 117 eyes (59.1%), while 81 eyes (40.9%) had a BCVA of 0.1 or higher. One week following 25G minimally invasive PPV, BCVA remained below 0.1 in 72 eyes (36.4%), and reached 0.1 or higher in 126 eyes (63.6%). McNemar’s test revealed a statistically significant improvement in categorical BCVA distribution from preoperative baseline to postoperative assessment (*p* < 0.001). Specifically, 52 eyes improved from BCVA < 0.1 to ≥ 0.1, whereas 7 eyes deteriorated from BCVA ≥ 0.1 to < 0.1.

### 3.4 Univariate analysis of factors influencing postoperative visual acuity

The patients were classified into two groups based on postoperative visual outcomes: the better-visual-acuity group, comprising individuals with a BCVA of 0.1 or higher, and the poorer-visual-acuity group, comprising those with a BCVA less than 0.1. Statistically significant differences between the two groups were identified in preoperative BCVA, maximum diameter of the foreign body, presence of vitreous hemorrhage, retinal detachment, endophthalmitis, and the location of the IOFB (*p* < 0.05). Conversely, no statistically significant differences were observed in demographic variables including gender and age, clinical factors such as time from injury to presentation and location of ocular trauma, characteristics of the foreign body (including its nature, entry site, and quantity), presence of traumatic cataract, or type of postoperative intraocular tamponade used (*p* > 0.05), as detailed in Table 2.

**Table 2:** Univariate analysis of factors influencing best-corrected visual acuity (BCVA) at 1 week postoperatively.

Characteristics	BCVA < 0.1 (*n* = 88)	BCVA≥0.1 (*n* = 110)	*X* ^2^	*p*
Gender			#	0.481
Male	83	105		
Female	5	5		
Age			3.649	0.161
< 18	6	4		
18 ~ 60	73	101		
> 60	9	5		
Time of visit			0.703	0.704
< 24 h	54	64		
24 ~ 72 h	18	28		
> 72 h	16	18		
Preoperative vision			81.315	< 0.001*
< 0.1	83	34		
≥0.1	5	76		
Wound location			1.849	0.397
Zone I	66	91		
Zone II	13	12		
Zone III	9	7		
IOFB diameter			13.943	< 0.001*
<3 mm	27	63		
≥3 mm	61	47		
IOFB location			23.143	< 0.001*
Macula	21	2		
Outside the	67	108		
Nature of IOFB			0.277	0.599
Non-magnetic	27	30		
Magnetic	61	80		
Traumatic cataracts			1.101	0.294
No	14	24		
Yes	74	86		
Vitreous hemorrhage			12.650	< 0.001*
No	45	83		
Yes	43	27		
Retinal detachment			18.080	< 0.001*
No	55	97		
Yes	33	13		
Endophthalmitis			12.052	0.001 *
No	70	105		
Yes	18	5		
Postoperative fillers			0.355	0.837
Silicone oil	66	80		
Gas	11	17		
Perfusate	11	13		
Number of IOFB				
= 1	78	104	2.298	0.130
> 1	10	6		

The asterisk (*) denotes statistical significance determined through univariate analysis; the number sign (#) signifies employment of Fisher’s exact test.

### 3.5 Multivariate logistic regression analysis of factors potentially influencing postoperative visual acuity

To identify independent risk factors for poor postoperative BCVA (BCVA < 0.1), a multivariate logistic regression model was constructed. After adjusting for potential confounding variables including age, gender, and foreign body location, three independent risk factors were identified: preoperative BCVA < 0.1, macular-located foreign body, and endophthalmitis. (Table 3).

**Table 3:** Multivariable logistic regression analysis of factors influencing best-corrected visual acuity (BCVA) at 1 week postoperatively.

Factor	B	S. E.	Wald	*p*	OR	95% CI for OR	
						Lower	Upper
Preoperative vision < 0.1	2.180	0.469	21.617	<0.001	8.849	3.530	22.185
IOFB diameter ≥3 mm	0.466	0.394	1.398	0.237	1.593	0.736	3.449
Macular IOFB	1.310	0.587	4.983	0.026	3.705	1.173	11.697
Retinal detachment	0.818	0.432	3.584	0.058	2.267	0.972	5.290
Endophthalmitis	1.141	0.535	4.546	0.033	3.131	1.097	8.938
Vitreous hemorrhage	0.106	0.394	0.072	0.788	1.112	0.514	2.406
Constant term	−1.846	0.874	4.459	0.035	0.158		

Three independent risk factors for poor postoperative BCVA (BCVA < 0.1) were identified. Preoperative BCVA < 0.1 demonstrated the strongest association (OR = 8.849, 95% CI: 3.530–22.185; *p* < 0.001), conferring an approximately 8.85-fold increased risk relative to patients with preoperative BCVA ≥ 0.1. Macular involvement of the foreign body was the second strongest predictor (OR = 3.705, 95% CI: 1.173–11.697; *p* = 0.025), associated with a ~ 3.7-fold elevated risk. Endophthalmitis was the third independent predictor (OR = 3.131, 95% CI: 1.097–8.938; *p* = 0.033), corresponding to a ~ 3.13-fold higher risk.

### 3.6 Predictive Modeling of postoperative visual acuity and ROC analysis

**Figure 1:**
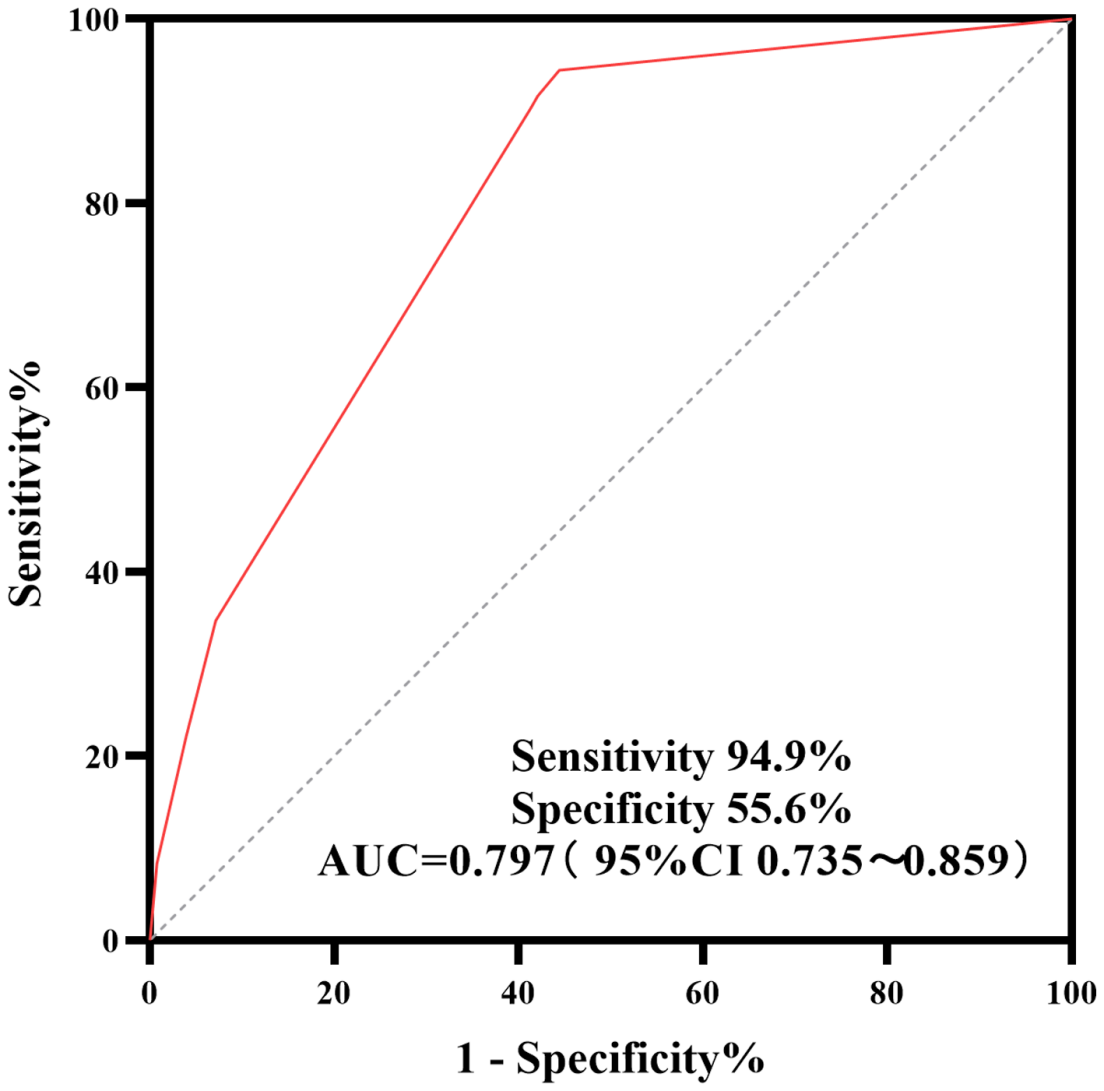
ROC curve for the prediction of poor postoperative visual outcome (BCVA < 0.1).

A multivariable logistic regression prediction model was developed based on independent predictors identified through multivariable analysis. The model estimates the probability of poor postoperative BCVA, with the linear predictor (L) defined as: L = Preoperative BCVA + 0.601 × (macular foreign body) + 0.523 × (endophthalmitis). The predicted probabilities derived from this model were used as the test variable, with the dichotomized postoperative BCVA outcome (<0.1 vs. ≥0.1) as the state variable, to generate the ROC curve (Figure 1). ROC curve analysis revealed that the combined predictor had an AUC of 0.797 (95% CI: 0.735–0.859), with an optimal cutoff value of 0.261 (derived from the maximum Youden’s index of 0.500) corresponding to a sensitivity of 94.4% and a specificity of 55.6%.

## 4 Discussion

IOFBs represent a distinct and severe form of open globe injury, associated with a high risk of visual impairment or blindness. This condition not only significantly compromises the occupational and functional capacity of affected individuals but also imposes substantial physical, psychological, and socioeconomic burdens on patients, their families, and society as a whole. Epidemiological studies (3–5) have consistently demonstrated that the majority of IOFBs are of metallic origin, and the affected population predominantly comprises young and middle-aged adults. The incidence is closely correlated with the level of occupational exposure. The findings of this study indicate that 91.4% of cases occur among young and middle-aged adults, and 59% of the identified foreign bodies are metallic in nature. These results are in strong agreement with those reported in the existing literature. These observations highlight the critical need for enhancing awareness regarding ocular protection in occupational settings. The implementation of standardized protective eyewear protocols is strongly recommended as an effective strategy to reduce the incidence of ocular trauma.

The 25G vitrectomy system, due to its distinctive advantages, has become a widely accepted therapeutic modality for a range of vitreoretinal disorders, such as diabetic retinopathy, epiretinal membrane, and vitreous hemorrhage. The system’s cannula-based incision design obviates the need for conjunctival and scleral suturing, thereby significantly reducing iatrogenic trauma associated with repeated instrument entry and withdrawal. This leads to a decreased incidence of postoperative complications, including retinal breaks, retinal detachment, and endophthalmitis (6). Under direct visualization, this procedure enables the concurrent achievement of three key therapeutic goals: removal of vitreous opacities to restore optical clarity; release of vitreoretinal traction to prevent retinal detachment; and elimination of intraocular pathogens to improve visual function (7, 8). Given these technical benefits combined with advances in microsurgical technology, the 25G system is now recognized as the preferred approach for the extraction of IOFBs (9, 10). In this study, 25G minimally invasive vitrectomy was performed on 198 patients with penetrating ocular injuries complicated by IOFB. The results revealed a 100% success rate in foreign body removal. Preoperatively, 81 eyes (40.9%) had a BCVA of ≥ 0.1, which increased to 126 eyes (63.6%) postoperatively. These findings provide strong evidence supporting the effectiveness of 25G vitrectomy in improving visual outcomes and reaffirm its critical role in the treatment of IOFBs.

Numerous studies have demonstrated that an initial visual acuity of less than 0.1 is a significant prognostic factor for unfavorable visual outcomes in patients with IOFBs (11, 12). Initial visual acuity may reflect the extent and severity of ocular damage, particularly when categorized as no light perception, which often indicates extensive structural injury involving the retina or optic nerve. These injuries typically present major challenges for surgical intervention and are commonly associated with limited visual recovery. This study corroborates previous findings by confirming that initial visual acuity independently predicts postoperative visual prognosis in patients with IOFBs.

Additionally, a statistically significant positive correlation has been identified between the size of the foreign body and the risk of retinal detachment (13, 14). From a mechanistic standpoint, larger foreign bodies tend to carry greater kinetic energy upon impact. Upon penetrating the globe, they not only create an entry wound but also induce scleral deformation, choroidal rupture, and irreversible damage to the photoreceptor layer. In some cases, the actual area of tissue destruction can extend up to two to three times the volume of the foreign body itself (15, 16). Univariate analysis suggested that a foreign body diameter ≥ 3 mm might be associated with an increased risk of poor visual outcomes. However, this association did not persist in multivariate regression analysis (*p* = 0.417), indicating that it may not be an independent predictor. The lack of statistical significance in the multivariate model could be attributed to several methodological limitations. First, the retrospective nature of the study introduces potential selection bias and increases heterogeneity among the study population. Second, the relatively small sample size (*n* = 198) likely limited the statistical power to detect subtle associations. To address these limitations, future research should adopt a standardized system for quantifying foreign body dimensions and incorporate more precise volumetric measurements. Furthermore, multi-center prospective cohort studies employing stratified analytical methods are recommended to better elucidate the dose–response relationship between foreign body characteristics and visual prognosis.

The retina functions as the critical neurosensory tissue responsible for transducing light stimuli into neural signals, thereby serving as a central component in visual perception. Preservation of retinal integrity is therefore essential for maintaining optimal visual function. Retinal detachment may initiate the pathogenic process of proliferative vitreoretinopathy (PVR), frequently leading to irreversible structural and functional impairment of the retinal neuroepithelium (17, 18) of particular clinical relevance is the fact that in cases involving intraocular foreign bodies combined with retinal detachment, PVR not only impairs intraoperative visualization and surgical accuracy but also significantly elevates the risk of recurrent retinal detachment (19). Univariate analysis in this study indicated an association between retinal detachment and poor postoperative visual outcomes. However, after adjusting for confounding factors including endophthalmitis and intraocular foreign body location in the multivariable analysis, this association was no longer statistically significant (*p* = 0.058). This suggests that the apparent prognostic effect of retinal detachment may be intertwined with factors such as intraocular inflammation severity and extent of foreign body-induced damage. Although not achieving conventional statistical significance, the borderline *p*-value suggests retinal detachment may still represent a clinically relevant negative predictor, a finding consistent with multicenter studies by Yang et al. (4), Nicoară et al. (17), and Lu et al. (20). Consequently, preoperative retinal detachment warrants appropriate clinical consideration in management decisions.

Numerous studies have demonstrated that the presence of endophthalmitis is an independent risk factor for poor visual prognosis (4, 21, 22). The findings of this retrospective analysis further corroborate this association, revealing that patients with endophthalmitis face a significantly higher likelihood of unfavorable visual outcomes. Pathophysiologically, the progression of endophthalmitis is characterized by increasing vitreous opacity, the accumulation of fibrous exudates and purulent material within the vitreous cavity, and concurrent retinal edema, necrosis, as well as the formation of fibrovascular membranes. These cumulative pathological processes not only complicate therapeutic interventions but also lead to a marked deterioration in visual prognosis.

From an epidemiological perspective, the incidence of endophthalmitis following open ocular trauma has been reported to range between 3.6 and 24% in prior studies (23–25). In the present study, 23 cases (11.6%) were diagnosed with post-traumatic endophthalmitis, which is consistent with the existing literature and represents a relatively low incidence rate. This favorable outcome may be partially attributed to the hospital’s location as a tertiary care center in an urban setting. Notably, 76.8% (152 out of 198) of patients presented within 72 h of injury, and 59.6% (118 cases) received standardized antimicrobial therapy and surgical debridement within 24 h, which likely played a crucial role in minimizing the risk of secondary infection. Based on these observations, we advocate for the implementation of a structured emergency protocol for managing open ocular trauma in clinical practice. Prompt identification of endophthalmitis, appropriate administration of broad-spectrum antibiotics, and timely performance of vitrectomy are critical interventions that should be prioritized to preserve residual vision and enhance long-term patient outcomes.

The macular region is located in the central portion of the retina, which forms the innermost layer of the posterior segment of the eyeball wall. It is the anatomical site responsible for the highest visual acuity. When intraocular foreign bodies involve the macular region, they can result in a substantial decline in central vision and may progress to permanent blindness. Valmaggia et al. (26) have demonstrated that the presence of foreign bodies in this region is an independent risk factor for poor visual recovery.

The underlying pathophysiological mechanism involves a complex, multi-dimensional cascade of events. Direct mechanical trauma caused by the foreign body can lead to structural disruption of the cone photoreceptor layer in the fovea centralis and rupture of Bruch’s membrane, resulting in irreversible visual loss. Furthermore, prolonged intraocular retention of the foreign body may trigger secondary pathological processes, including macular cystoid edema and choroidal neovascularization. In addition, due to the delicate nature of macular tissue and the limited surgical access in this region, the risk of iatrogenic retinal tears and the formation of epiretinal membranes during intervention is notably elevated. Collectively, these factors contribute to a consistently poor visual prognosis (27–29). The findings of this study corroborate previous reports, reinforcing the conclusion that IOFBs involving the macular region are independently associated with adverse visual outcomes. Therefore, in clinical settings, the identification of a macular-involved IOFB necessitates urgent evaluation and management. Immediate medical intervention should be implemented, and surgical procedures must be conducted with meticulous precision to minimize complications and improve long-term visual outcomes.

Our study investigated prognostic factors for visual outcomes following minimally invasive vitrectomy. Crucially, visual prognosis is governed not by surgical technique alone, but by the interplay between the inherent severity of the primary trauma and the procedural intervention—where trauma severity serves as the dominant, non-modifiable prognostic anchor. Within this framework, wound location (Zone I vs. Zone III) emerged as a consistently robust and statistically independent predictor of both postoperative astigmatism and cicatricial remodeling, with effects persisting across all vitrectomy platforms evaluated. Zone I (central corneal) injuries directly compromise the optical surface architecture, resulting in high-magnitude, topographically irregular refractive error that is frequently resistant to spectacle or contact lens correction. In contrast, Zone III (posterior scleral) wounds are strongly associated with dysregulated wound healing and early PVR activation, thereby substantially increasing the incidence of tractional retinal detachment and other vision-impairing cicatricial sequelae (30, 31). While our multivariable logistic regression model rigorously adjusted for established clinical covariates—including foreign body location, endophthalmitis, and preoperative BCVA—the absence of standardized, quantitative metrics for primary wound extent (e.g., linear dimension, tissue defect volume, and zone-specific depth) represents a methodological limitation that may attenuate the precision of effect estimates. Accordingly, future prospective studies should incorporate standardized, quantitative assessments of primary wound characteristics—including size, anatomical location, and volumetric tissue defect—to rigorously isolate the independent contribution of surgical technique to visual prognosis.

In light of its retrospective design, this study has several inherent limitations that merit careful consideration. First, the lack of randomization and the involvement of multiple surgeons in treatment decision-making may introduce unmeasured confounding and inter-operator variability, potentially affecting the internal validity of efficacy assessments. Second, the one-week postoperative follow-up interval is insufficient to capture the full trajectory of visual recovery or assess definitive functional outcomes following vitrectomy. Notwithstanding these constraints, the primary aim of this study was to identify modifiable and non-modifiable independent predictors of early postoperative visual acuity—and to develop and internally validate a clinically applicable predictive model for timely risk stratification and targeted intervention in high-risk patients. To strengthen causal inference and generalize findings, future work should prioritize prospective, multicenter cohort studies with standardized outcome adjudication, extended longitudinal follow-up, and rigorous adjustment for wound-specific anatomical and quantitative parameters—ultimately advancing evidence-based management and long-term visual rehabilitation in patients with intraocular foreign bodies.

## Data Availability

The raw data supporting the conclusions of this article will be made available by the authors, without undue reservation.
